# P-1845. Evaluation of the immunogenicity and protective efficacy of a fusion vaccine based on the class 5a fimbrial tip of Enterotoxigenic *Escherichia coli* (ETEC) in an *Aotus nancymaae* CS14+ and CFA/I+ challenge model

**DOI:** 10.1093/ofid/ofae631.2006

**Published:** 2025-01-29

**Authors:** Juan Carlos Gaspar, Tyler Moeller, Michael Prouty, Rosa Nunez, Nereyda Espinoza, Monica Nieto

**Affiliations:** Naval Medical Research SOUTH, Lima, Lima, Peru; Naval Medical Research Unit 6, Maryland, Maryland; U. S. Naval Medical Research Unit SOUTH, Washington, Washington; Naval Medical Research Unit 6, Lima, Lima, Peru; Naval Medical Research Unit 6, Lima, Lima, Peru; Naval Medical Research Unit 6, Lima, Lima, Peru

## Abstract

**Background:**

Enterotoxigenic *Escherichia coli* (ETEC) colonizes intestinal epithelial cells via colonization factors (CFs), fimbrial, non-fimbrial, and helical adhesins. Once attached, ETEC can release enterotoxins including heat-stable toxin (ST) and/or heat-labile toxin (LT) which cause hypersecretion of fluid in the intestinal lumen resulting in watery diarrhea. Currently, there are no effective countermeasures against ETEC, and antibiotic treatments are losing effectiveness due to increasing antimicrobial resistance. Passive or active immunization against CFs are alluring targets to prevent adhesion, colonization, and ultimately, diarrhea. However, ETEC which infects humans exhibits remarkable heterogeneity and expresses at least 29 immunologically different CFs, making it difficult to create a broadly targeting vaccine that is effective in preventing diarrhea caused by multiple ETEC strains. One third of the CFs that have been described belong to one of three phylogenetic families: CFA/I-like family, which is defined as Class 5 fimbriae divided into three subclasses, 5a, 5b and 5c that are highly related in structure and function.

**Methods:**

In this work, we evaluated the efficacy of a vaccine based on the CFA/I adhesin-pilin fusion (CfaEB) fused to the pilins of colonization factor CS14 (CsuA2) and CS4 (CsfA) to generate broad cross-protection against members of subclass 5a. We previously established a non-human primate challenge model for CS14^+^ ETEC (strain WS3294A) and found that a 5x10^12^ CFU dose was needed to achieve a sufficiently high (70%) attack rate. Therefore, the same challenge dose was used to evaluate the effectiveness of the vaccine candidate CfaEp3 (CfaEB-CsuA2-CsfA) against ETEC H10407 (CFA/I; n=10) and WS3294A (CS14; n=10).

**Results:**

Animals that received four vaccine doses with dmLT adjuvant, administered intradermally, were protected from challenge with WS3294A (91% efficacy; *p*=0.05). However, only 20% protection against ETEC H10407 was achieved.

**Conclusion:**

Although the vaccine candidate did not present cross-protection against members of subclass 5a, the model opens the possibility of future testing of combined vaccines to combat infection with the main pathogenic strains of ETEC encountered by travelers, the military, and civilian populations.

**Disclosures:**

**All Authors**: No reported disclosures

